# Poly[bis(phenethyl­ammonium) [di­bromido­plumbate(II)]-di-μ-bromido]]

**DOI:** 10.1107/S160053680903712X

**Published:** 2009-10-07

**Authors:** Kengo Shibuya, Masanori Koshimizu, Fumihiko Nishikido, Haruo Saito, Shunji Kishimoto

**Affiliations:** a Institute of Physics, Graduate School of Arts and Sciences, University of Tokyo, 3-8-1 Komaba, Meguro-ku, Tokyo 153-8902, Japan; b CREST, Japan Science and Technology Agency, 5 Sanbancho, Chiyoda-ku, Tokyo 102-0075, Japan; c Department of Applied Chemistry, Tohoku University, Graduate School of Engineering, 6-6-07 Aoba, Aramaki, Aoba-ku, Sendai 980-8579, Japan; d Molecular Imaging Center, National Institute of Radiological Sciences, 4-9-1 Anagawa, Inage-ku, Chiba 263-8555, Japan; e Institute of Materials Structure Science, High Energy Accelerator Research Organization, 1-1 Oho, Tukuba 305-0801, Japan

## Abstract

Crystals of the title compound, {(C_6_H_5_C_2_H_4_NH_3_)_2_[PbBr_4_]}_*n*_, were grown at room temperature from a solution in *N*,*N*-dimethyl­formamide (DMF) using nitro­methane as the poor solvent. This perovskite-type organic–inorganic hybrid compound consists of well ordered sheets of corner-sharing disordered PbBr_6_ octa­hedra separated by bilayers of phenethyl­ammonium cations. The octa­hedra are rotated and tilted due to N—H⋯Br hydrogen bonds with the ammonium groups, generating a superstructure in the unit cell similar to that of the tetra­chloridoplumbate (C_6_H_5_C_2_H_4_NH_3_)_2_[PbCl_4_].

## Related literature

The title compound has been studied previously and the lattice parameters reported without the complete structure (Mitzi, 1999 ▶). The optical characteristics have been investigated using thin films, see: Cheng *et al.* (2005 ▶); Kitazawa & Watanabe (2005 ▶). Promising applications have been reported on electroluminescent devices and scintillators, see: Era *et al.* (1995 ▶); Kishimoto *et al.* (2008 ▶); van der Eijk *et al.* (2008 ▶). Structural data of some related materials have been published; for (C_6_H_5_C_2_H_4_NH_3_)_2_PbCl_4_, see: Mitzi (1999 ▶); for (C_6_H_5_C_2_H_4_NH_3_)_2_CuBr_4_, see: Willett (1990 ▶); for (C_6_H_5_C_2_H_4_NH_3_)_2_ZnBr_4_, see: Huh *et al.* (2006 ▶); for (C_6_H_5_C_2_H_4_NH_3_)PbBr_3_, see: Billing & Lemmerer (2003 ▶). For van der Waals radii, see: Bondi (1964 ▶). For halogen hydrogen bonding, see: Chapuis *et al.* (1976 ▶). 
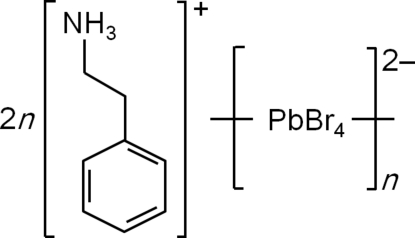


## Experimental

### 

#### Crystal data

(C_8_H_12_N)_2_[PbBr_4_]
*M* *_r_* = 771.20
Triclinic, 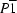

*a* = 11.6150 (4) Å
*b* = 11.6275 (5) Å
*c* = 17.5751 (6) Å
α = 99.5472 (12)°
β = 105.7245 (10)°
γ = 89.9770 (12)°
*V* = 2250.62 (15) Å^3^
*Z* = 4
Mo *K*α radiation
μ = 14.63 mm^−1^
*T* = 296 K
0.25 × 0.20 × 0.03 mm

#### Data collection

Rigaku R-AXIS RAPID diffractometer
Absorption correction: numerical (*ABSCOR*; Higashi, 1999 ▶) *T*_min_ = 0.106, *T*_max_ = 0.645
20072 measured reflections
10077 independent reflections
7157 reflections with *I* > 2σ(*I*)
*R*_int_ = 0.053

#### Refinement

*R*[*F* ^2^ > 2σ(*F* ^2^)] = 0.042
*wR*(*F* ^2^) = 0.106
*S* = 0.97
10077 reflections
416 parameters
H-atom parameters constrained
Δρ_max_ = 3.26 e Å^−3^
Δρ_min_ = −2.53 e Å^−3^

### 

Data collection: *PROCESS-AUTO* (Rigaku Corporation, 1998 ▶); cell refinement: *PROCESS-AUTO*; data reduction: *CrystalStructure* (Rigaku Americas & Rigaku Corporation, 2008 ▶); program(s) used to solve structure: *SIR2002* (Burla *et al.*, 2003 ▶); program(s) used to refine structure: *SHELXL97* (Sheldrick, 2008 ▶); molecular graphics: *CrystalMaker* (Palmer, 2009 ▶); software used to prepare material for publication: *publCIF* (Westrip, 2009 ▶).

## Supplementary Material

Crystal structure: contains datablocks global, I. DOI: 10.1107/S160053680903712X/zq2004sup1.cif

Structure factors: contains datablocks I. DOI: 10.1107/S160053680903712X/zq2004Isup2.hkl

Additional supplementary materials:  crystallographic information; 3D view; checkCIF report

## Figures and Tables

**Table 1:** Selected geometric parameters (Å, °)

Pb1—Br3	2.8786 (8)
Pb1—Br4	2.9927 (7)
Pb1—Br1	2.9957 (7)
Pb1—Br6	3.0080 (7)
Pb1—Br5	3.0095 (7)
Pb1—Br2	3.1965 (8)
Pb2—Br8	2.8755 (8)
Pb2—Br5^i^	2.9935 (6)
Pb2—Br6	2.9957 (7)
Pb2—Br1^ii^	3.0082 (7)
Pb2—Br4^iii^	3.0110 (7)
Pb2—Br7	3.1982 (8)

Symmetry codes: (i) 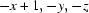; (ii) 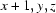; (iii) 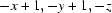.

**Table 2:** Hydrogen-bond geometry (Å, °)

*D*—H⋯*A*	*D*—H	H⋯*A*	*D*⋯*A*	*D*—H⋯*A*
N1—H1⋯Br1	0.89	3.18	3.508 (5)	104
N1—H3⋯Br2	0.89	2.54	3.411 (5)	165
N2—H13⋯Br6	0.89	3.17	3.509 (5)	105
N2—H14⋯Br7	0.89	2.54	3.416 (5)	167
N3—H26⋯Br7	0.89	2.71	3.448 (6)	142
N3—H27⋯Br2	0.89	2.62	3.486 (6)	164
N4—H37⋯Br4	0.89	2.68	3.465 (5)	148
N4—H39⋯Br2	0.89	2.73	3.462 (6)	140

**Fig. 1.**
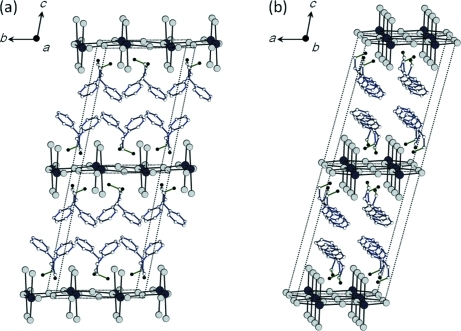
Packing diagram of (C6H5C2H4NH3)2PbBr4, approximately viewed down (a) the a axis and (b) the b axis. Dashed line shows the outline of two triclinic unit cells along with the c axis. For clarity, the atoms are represented as spheres with each uniform size for the PbBr6 octahedra and the phenethylammonium, respectively. Hydrogen atoms are omitted.

**Fig. 2.**
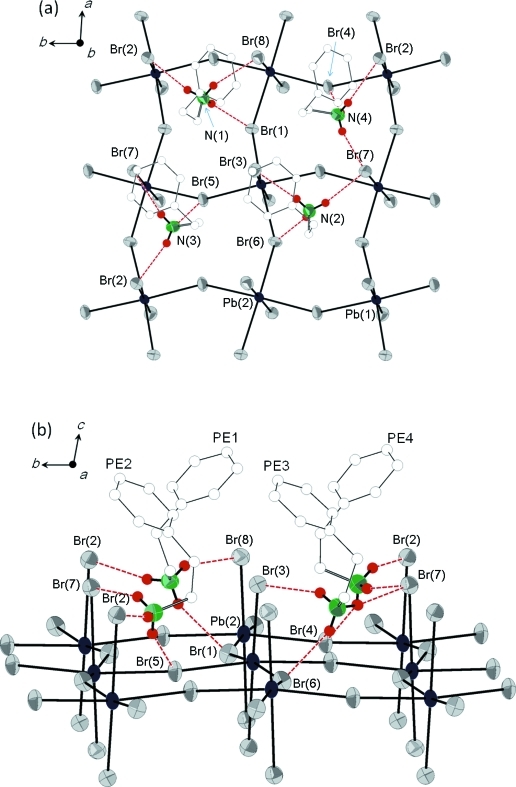
The relative rotation of PbBr6 due to hydrogen bonding (dashed lines) between the octahedra and ammonium groups. The structure is approximately viewed down (a) the b axis and (b) the a axis. The thermal ellipsoids are drawn at 50% probability for nitrogen, bromine, and lead atoms. The hydrogen atoms of nothing to do with hydrogen bonding are omitted.

## supplementary crystallographic information

### Comment

Recently, much attention has been paid to low-dimensional materials that often exhibit characteristic electronic properties considerably different from those of bulk ones. However, their crystallographic studies are limited because their anisotropic growth nature makes it difficult to obtain a good single crystal. Mitzi reported the structure of the tetrachloroplumbate, (C_6_H_5_C_2_H_4_NH_3_)_2_PbCl_4_, whose single crystals required approximately one year to be grown up. The present paper is the first report of the detailed structure of the tetrabromoplumbate, whose single crystals were grown up in approximately two months; in order to compare with some related materials: see the tetrachloroplumbate, the tetrabromozincate, (C_6_H_5_C_2_H_4_NH_3_)_2_ZnBr_4_ (Huh *et al.*, 2006), and the tribromoplumbate, (C_6_H_5_C_2_H_4_NH_3_)PbBr_3_ (Billing & Lemmerer, 2003).

Fig. 1 shows the packing diagram of (C_6_H_5_C_2_H_4_NH_3_)_2_PbBr_4_, viewed approximately along the *c* axis. The sheets of corner-sharing PbBr_6_ octahedra are separated by bilayers of phenethylammonium cations. The corner-sharing PbBr_6_ octahedra are the common structure among bis-(phenethylammonium) tetrahaloplumbates, (C_6_H_5_C_2_H_4_NH_3_)_2_Pb*X*_4_ (*X *= Cl, Br, and I), regardless of the halogen, but are different from face-sharing PbBr_6_ octahedra of the tribromoplumbate, (C_6_H_5_C_2_H_4_NH_3_)PbBr_3_, and from isolated tetrahedral ZnBr_4_ of the tetrabromozincate, (C_6_H_5_C_2_H_4_NH_3_)_2_ZnBr_4_. As the structure of halometalate is notably controlled by surrounding organic molecules, hydrogen bondings between them are discussed later.

Dashed line in Fig. 1 displays the triclinic unit cell, which is similar to the triclinic unit cell of the tetrachloroplumbate, (C_6_H_5_C_2_H_4_NH_3_)_2_PbCl_4_, but different from the monoclinic unit cell of the tetraiodoplumbate, (C_6_H_5_C_2_H_4_NH_3_)_2_PbI_4_. The present tetrabromoplumbate possesses two independent but similar Pb atoms with distorted octahedral coordination. The Pb—Br bond lengths range from 2.8755 (8) to 3.1982 (8) Å (average: 3.0136 (7) Å) and Br—Pb—Br bond angles range from 83.44 (2)° to 96.67 (2)° and from 170.97 (2)° to 179.36 (2)°. These angles are somewhat different from those of the perfect octahedron, *i.e.*, 90.0° and 180°, respectively. Furthermore, the bridging Pb—Br—Pb bond angles significantly differ from 180° and range from 150.77 (3)° to 152.15 (3)°. This indicates that adjacent PbBr_6_ are rotated relative to each other.

Fig. 2 shows the relative rotation of PbBr_6_ in the sheet and the hydrogen bondings between the octahedra and ammonium groups. Each ammonium group interacts with three halogen anions through N—H···Br hydrogen bonding in "terminal halogen configuration" involving two terminal halogen anions and one bridging halogen anion (Chapuis *et al.*, 1976). The average hydrogen-bonding distance is 2.630 (2) Å, which is considerably shorter than the sum of the van der Waals radii for H (1.20–1.45 Å) and Br (1.95 Å) (Bondi, 1964). As a result, the opposite sides of the quadrangle, defined by one set of four PbBr_6_ octahedra, are "pinched-in" or "pushed-out" as shown in Fig. 2. In addition, there are four independent phenethylammonium depicted as PE1, PE2, PE3, and PE4, having similar bond lengths and bond angles. Therefore, two sides of the unit cell along with *a* and *b* axes are about twice length of a PbBr_6_ to have a superstructure in it.

There is no significant π-π interaction found in the organic bilayers because the adjacent aromatic rings are considerably separated by centroid-to-centroid distance of 5.748 (9) Å between PE1 and PE4, and 5.787 (9) Å between PE2 and PE3, respectively. The van der Waals radius for aromatic carbon atoms is about 1.77 Å (Bondi, 1964).

### Experimental

Single crystals were obtained in the following three steps. First, phenethylamine bromide, C_6_H_5_C_2_H_2_NH_3_Br, as the precursor was synthesized at 10 C° from stoichiometric amount of hydrobromic acid, HBr, and phenethylamine, C_6_H_5_C_2_H_2_NH_2_, by their acid-base reaction in a flask. After evaporating the solvent, water, at 70 C°, the white deposition was washed by diethyl ether to remove unreacted reagents and dried in vacuum. Second, the objective compound was synthesized at 25 C° in dry nitrogen atmosphere from stoichiometric amount of the precursor and lead bromide (II), PbBr_2_, using dehydrated *N*,*N*'-dimethylformamide (DMF) as a good solvent. The purity of PbBr_2_ powder was 4 N, and it was used as delivered from Kojundo Chemical Laboratory Co., Japan. Third, the solution was filtered and contained in a glass bottle for the crystal growth. The bottle was contained in a shaded desiccator where another bottle with nitromethane as a poor solvent was also contained. Then, the vapor of the poor solvent was gradually diffused into the solution to reduce the solubility. Settling it two months grew colorless transparent crystals at the bottom of the former bottle. The crystal size was typically 8 mm × 6 mm × 1 mm, and the one used for the crystallographic study was 0.25 mm × 0.20 mm × 0.03 mm.

### Refinement

The structure was solved by direct methods and expanded using Fourier techniques. The non-hydrogen atoms were refined anisotropically. Hydrogen atoms were refined using the riding model. The final cycle of full-matrix least-squares refinement on F^2^ was based on 10107 observed reflections and 416 variable parameters and converged (largest parameter shift was 0.00 times its e.s.d.) with unweighted and weighted agreement factors of R1 = 0.0460 and wR2 = 0.1483. The standard deviation of an observation of unit weight was 1.06. Unit weights were used. The maximum and minimum peaks on the final difference Fourier map corresponded to 3.95 and -2.77 e^-^/Å^3^, respectively.

### Crystal data

**Table:**

(C_8_H_12_N)_2_[PbBr_4_]	*Z* = 4
*M**_r_* = 771.20	*F*(000) = 1424.00
Triclinic, *P*1	*D*_x_ = 2.276 Mg m^−^^3^
Hall symbol: -P 1	Mo *K*α radiation, λ = 0.71075 Å
*a* = 11.6150 (4) Å	Cell parameters from 15239 reflections
*b* = 11.6275 (5) Å	θ = 3.2–27.5°
*c* = 17.5751 (6) Å	µ = 14.63 mm^−^^1^
α = 99.5472 (12)°	*T* = 296 K
β = 105.7245 (10)°	Platelet, colourless
γ = 89.9770 (12)°	0.25 × 0.20 × 0.03 mm
*V* = 2250.62 (15) Å^3^	

### Data collection

**Table:**

Rigaku R-AXIS RAPID diffractometer	7157 reflections with *I* > 2σ(*I*)
Detector resolution: 10.00 pixels mm^-1^	*R*_int_ = 0.053
ω scans	θ_max_ = 27.5°
Absorption correction: numerical see: Higashi (1999)	*h* = −15→12
*T*_min_ = 0.106, *T*_max_ = 0.645	*k* = −15→15
20072 measured reflections	*l* = −22→22
10077 independent reflections	

### Refinement

**Table:**

Refinement on *F*^2^	0 restraints
*R*[*F*^2^ > 2σ(*F*^2^)] = 0.042	H-atom parameters constrained
*wR*(*F*^2^) = 0.106	*w* = 1/[σ^2^(*F*_o_^2^) + (0.0442*P*)^2^] where *P* = (*F*_o_^2^ + 2*F*_c_^2^)/3
*S* = 0.97	(Δ/σ)_max_ < 0.001
10077 reflections	Δρ_max_ = 3.26 e Å^−^^3^
416 parameters	Δρ_min_ = −2.53 e Å^−^^3^

### Special details

**Table:**

Geometry. ENTER SPECIAL DETAILS OF THE MOLECULAR GEOMETRY
Refinement. Refinement was performed using all reflections. The weighted *R*-factor (*wR*) and goodness of fit (*S*) are based on *F*^2^. *R*-factor (gt) are based on *F*. The threshold expression of *F*^2^ > 2.0 σ(*F*^2^) is used only for calculating *R*-factor (gt).

### Fractional atomic coordinates and isotropic or equivalent isotropic displacement parameters (Å^2^)

**Table:**

	*x*	*y*	*z*	*U*_iso_*/*U*_eq_	
Pb1	0.24494 (2)	0.239633 (18)	−0.011473 (16)	0.03270 (15)	
Pb2	0.74492 (2)	0.254601 (18)	−0.011604 (16)	0.03274 (15)	
Br1	−0.00101 (6)	0.18802 (6)	0.00154 (5)	0.04426 (17)	
Br2	0.31464 (7)	0.27662 (6)	0.18009 (5)	0.04282 (16)	
Br3	0.18198 (7)	0.20941 (6)	−0.18389 (5)	0.04710 (18)	
Br4	0.18722 (7)	0.49165 (5)	−0.00100 (5)	0.04628 (18)	
Br5	0.31258 (7)	−0.00779 (5)	0.00106 (5)	0.04622 (18)	
Br6	0.49894 (6)	0.31257 (6)	0.00149 (5)	0.04407 (18)	
Br7	0.81414 (7)	0.31351 (6)	0.18005 (5)	0.04330 (17)	
Br8	0.68260 (7)	0.19889 (6)	−0.18383 (5)	0.04685 (17)	
N1	0.1391 (5)	0.0296 (4)	0.1519 (3)	0.0467 (14)	
N2	0.6387 (5)	0.5470 (4)	0.1515 (3)	0.0488 (15)	
N3	0.5666 (5)	0.1314 (4)	0.1523 (3)	0.0452 (14)	
N4	0.0652 (5)	0.4444 (4)	0.1512 (3)	0.0488 (15)	
C1	0.0360 (7)	0.0491 (7)	0.1886 (5)	0.057 (2)	
C2	0.0764 (7)	0.1128 (6)	0.2729 (5)	0.057 (2)	
C3	0.1601 (7)	0.0445 (6)	0.3284 (4)	0.0496 (19)	
C4	0.2817 (8)	0.0695 (7)	0.3537 (5)	0.061 (2)	
C5	0.3586 (10)	0.0075 (9)	0.4031 (6)	0.083 (3)	
C6	0.3178 (12)	−0.0828 (9)	0.4286 (5)	0.092 (3)	
C7	0.1951 (13)	−0.1109 (8)	0.4068 (6)	0.093 (3)	
C8	0.1187 (9)	−0.0466 (7)	0.3569 (5)	0.069 (2)	
C9	0.5357 (7)	0.5449 (7)	0.1881 (5)	0.056 (2)	
C10	0.5782 (7)	0.5256 (6)	0.2735 (4)	0.055 (2)	
C11	0.6609 (8)	0.6222 (6)	0.3296 (4)	0.052 (2)	
C12	0.6160 (9)	0.7258 (7)	0.3569 (5)	0.068 (2)	
C13	0.6902 (13)	0.8155 (8)	0.4068 (6)	0.090 (3)	
C14	0.8131 (12)	0.8005 (9)	0.4275 (5)	0.092 (3)	
C15	0.8591 (10)	0.6986 (9)	0.4008 (5)	0.080 (3)	
C16	0.7830 (9)	0.6098 (8)	0.3539 (5)	0.064 (2)	
C17	0.5851 (7)	0.0204 (6)	0.1850 (4)	0.057 (2)	
C18	0.5733 (8)	0.0381 (6)	0.2699 (4)	0.058 (2)	
C19	0.6595 (7)	0.1290 (6)	0.3289 (4)	0.0483 (19)	
C20	0.6198 (8)	0.2342 (7)	0.3589 (5)	0.063 (2)	
C21	0.6982 (12)	0.3185 (8)	0.4121 (6)	0.084 (3)	
C22	0.8178 (11)	0.2988 (9)	0.4339 (5)	0.085 (3)	
C23	0.8600 (9)	0.1948 (9)	0.4045 (5)	0.079 (2)	
C24	0.7809 (8)	0.1109 (8)	0.3529 (5)	0.063 (2)	
C25	0.0873 (7)	0.5722 (5)	0.1864 (5)	0.055 (2)	
C26	0.0736 (8)	0.5965 (6)	0.2690 (5)	0.061 (2)	
C27	0.1587 (8)	0.5336 (6)	0.3276 (4)	0.053 (2)	
C28	0.2787 (8)	0.5620 (8)	0.3514 (5)	0.067 (2)	
C29	0.3581 (9)	0.5032 (9)	0.4037 (6)	0.083 (3)	
C30	0.3166 (12)	0.4151 (9)	0.4318 (5)	0.094 (3)	
C31	0.1934 (12)	0.3866 (8)	0.4114 (6)	0.088 (3)	
C32	0.1190 (9)	0.4465 (7)	0.3589 (5)	0.071 (2)	
H1	0.1122	−0.0084	0.1017	0.056*	
H2	0.1928	−0.0124	0.1804	0.056*	
H3	0.1730	0.0983	0.1519	0.056*	
H4	−0.0232	0.0936	0.1568	0.069*	
H5	−0.0017	−0.0257	0.1878	0.069*	
H6	0.0068	0.1303	0.2925	0.068*	
H7	0.1164	0.1863	0.2737	0.068*	
H8	0.3126	0.1310	0.3363	0.073*	
H9	0.4401	0.0280	0.4193	0.100*	
H10	0.3710	−0.1266	0.4606	0.110*	
H11	0.1653	−0.1716	0.4255	0.111*	
H12	0.0369	−0.0653	0.3419	0.082*	
H13	0.6115	0.5585	0.1010	0.059*	
H14	0.6741	0.4792	0.1523	0.059*	
H15	0.6913	0.6046	0.1795	0.059*	
H16	0.4964	0.6184	0.1865	0.068*	
H17	0.4778	0.4830	0.1570	0.068*	
H18	0.6194	0.4531	0.2745	0.066*	
H19	0.5090	0.5168	0.2932	0.066*	
H20	0.5339	0.7351	0.3414	0.082*	
H21	0.6592	0.8845	0.4262	0.108*	
H22	0.8648	0.8611	0.4602	0.111*	
H23	0.9414	0.6899	0.4145	0.096*	
H24	0.8139	0.5390	0.3377	0.077*	
H25	0.5738	0.1194	0.1024	0.054*	
H26	0.6213	0.1856	0.1829	0.054*	
H27	0.4938	0.1555	0.1519	0.054*	
H28	0.6641	−0.0065	0.1848	0.068*	
H29	0.5263	−0.0390	0.1512	0.068*	
H30	0.4923	0.0601	0.2687	0.070*	
H31	0.5849	−0.0357	0.2891	0.070*	
H32	0.5387	0.2485	0.3430	0.075*	
H33	0.6700	0.3882	0.4331	0.101*	
H34	0.8713	0.3562	0.4688	0.102*	
H35	0.9414	0.1816	0.4196	0.094*	
H36	0.8092	0.0403	0.3336	0.076*	
H37	0.0738	0.4324	0.1018	0.059*	
H38	−0.0089	0.4220	0.1496	0.059*	
H39	0.1175	0.4033	0.1816	0.059*	
H40	0.1676	0.5967	0.1874	0.066*	
H41	0.0312	0.6171	0.1526	0.066*	
H42	−0.0078	0.5744	0.2670	0.073*	
H43	0.0858	0.6798	0.2886	0.073*	
H44	0.3076	0.6224	0.3321	0.080*	
H45	0.4395	0.5242	0.4194	0.099*	
H46	0.3702	0.3729	0.4649	0.113*	
H47	0.1636	0.3291	0.4329	0.105*	
H48	0.0373	0.4270	0.3438	0.086*	

### Atomic displacement parameters (Å^2^)

**Table:**

	*U*^11^	*U*^22^	*U*^33^	*U*^12^	*U*^13^	*U*^23^
Pb1	0.027 (12)	0.02690 (13)	0.04648 (18)	0.00509 (10)	0.01243 (12)	0.01035 (11)
Pb2	0.027 (12)	0.02591 (13)	0.04690 (18)	0.00448 (10)	0.01214 (12)	0.00715 (11)
Br1	0.0289 (3)	0.0460 (4)	0.0576 (5)	0.0041 (2)	0.0130 (3)	0.0062 (3)
Br2	0.0381 (4)	0.0421 (3)	0.0472 (4)	0.0046 (3)	0.0104 (3)	0.0071 (3)
Br3	0.0522 (4)	0.0434 (4)	0.0430 (4)	−0.0007 (3)	0.0087 (3)	0.0074 (3)
Br4	0.0558 (4)	0.0270 (3)	0.0602 (4)	0.0065 (3)	0.0206 (4)	0.0115 (3)
Br5	0.0557 (4)	0.0268 (3)	0.0604 (4)	0.0064 (3)	0.0212 (4)	0.0105 (3)
Br6	0.0276 (3)	0.0502 (4)	0.0577 (5)	0.0072 (3)	0.0131 (3)	0.0163 (3)
Br7	0.0384 (4)	0.0435 (3)	0.0482 (4)	0.0037 (3)	0.0105 (3)	0.0109 (3)
Br8	0.0509 (4)	0.0438 (4)	0.0432 (4)	0.0082 (3)	0.0077 (3)	0.0086 (3)
N1	0.053 (4)	0.043 (3)	0.041 (3)	0.010 (2)	0.006 (3)	0.007 (2)
N2	0.052 (4)	0.043 (3)	0.050 (4)	0.003 (2)	0.009 (3)	0.012 (2)
N3	0.036 (3)	0.053 (3)	0.048 (3)	−0.001 (2)	0.015 (3)	0.008 (2)
N4	0.044 (3)	0.051 (3)	0.054 (4)	0.009 (2)	0.016 (3)	0.011 (3)
C1	0.046 (4)	0.067 (5)	0.063 (5)	0.010 (4)	0.014 (4)	0.024 (4)
C2	0.060 (5)	0.062 (5)	0.056 (5)	0.013 (4)	0.027 (4)	0.012 (4)
C3	0.060 (5)	0.049 (4)	0.041 (4)	0.006 (3)	0.020 (4)	0.001 (3)
C4	0.068 (6)	0.064 (5)	0.051 (5)	0.000 (4)	0.016 (4)	0.009 (4)
C5	0.072 (7)	0.108 (8)	0.058 (6)	0.003 (6)	0.000 (5)	0.014 (5)
C6	0.118 (11)	0.090 (8)	0.042 (5)	0.026 (7)	−0.016 (6)	0.004 (5)
C7	0.163 (13)	0.055 (6)	0.056 (6)	−0.003 (6)	0.019 (7)	0.019 (4)
C8	0.085 (7)	0.067 (5)	0.055 (5)	−0.010 (4)	0.020 (5)	0.011 (4)
C9	0.040 (4)	0.061 (5)	0.060 (5)	0.005 (3)	0.008 (4)	−0.003 (4)
C10	0.052 (5)	0.066 (5)	0.050 (5)	−0.004 (4)	0.021 (4)	0.005 (4)
C11	0.064 (5)	0.054 (4)	0.047 (5)	0.010 (4)	0.024 (4)	0.018 (3)
C12	0.076 (7)	0.074 (6)	0.059 (6)	0.019 (5)	0.022 (5)	0.018 (4)
C13	0.145 (11)	0.058 (6)	0.062 (7)	0.020 (6)	0.023 (7)	0.005 (5)
C14	0.129 (12)	0.079 (7)	0.045 (5)	−0.028 (7)	−0.009 (6)	−0.002 (5)
C15	0.081 (8)	0.106 (8)	0.049 (6)	−0.006 (6)	0.016 (5)	0.011 (5)
C16	0.075 (7)	0.073 (6)	0.051 (5)	0.020 (5)	0.026 (5)	0.011 (4)
C17	0.066 (5)	0.041 (4)	0.060 (5)	0.013 (3)	0.007 (4)	0.015 (3)
C18	0.068 (6)	0.059 (5)	0.050 (5)	−0.003 (4)	0.011 (4)	0.024 (4)
C19	0.057 (5)	0.052 (4)	0.039 (4)	0.002 (3)	0.012 (4)	0.018 (3)
C20	0.066 (6)	0.070 (5)	0.054 (5)	0.014 (4)	0.017 (4)	0.017 (4)
C21	0.138 (11)	0.057 (6)	0.054 (6)	0.011 (6)	0.024 (7)	0.003 (4)
C22	0.108 (10)	0.087 (7)	0.043 (5)	−0.021 (6)	−0.006 (6)	0.006 (5)
C23	0.055 (6)	0.113 (8)	0.060 (6)	−0.007 (6)	0.007 (5)	0.007 (6)
C24	0.062 (6)	0.077 (6)	0.049 (5)	0.014 (4)	0.014 (4)	0.009 (4)
C25	0.057 (5)	0.037 (4)	0.064 (5)	−0.002 (3)	0.008 (4)	0.003 (3)
C26	0.067 (6)	0.048 (4)	0.061 (5)	0.017 (4)	0.012 (4)	−0.005 (4)
C27	0.068 (6)	0.047 (4)	0.048 (5)	0.005 (3)	0.029 (4)	0.000 (3)
C28	0.061 (6)	0.086 (6)	0.058 (5)	0.004 (4)	0.019 (5)	0.020 (4)
C29	0.065 (7)	0.113 (8)	0.059 (6)	0.003 (6)	0.007 (5)	0.003 (6)
C30	0.135 (11)	0.083 (7)	0.045 (6)	0.043 (7)	−0.002 (7)	0.004 (5)
C31	0.136 (11)	0.063 (6)	0.057 (6)	−0.005 (6)	0.014 (7)	0.011 (5)
C32	0.077 (7)	0.069 (6)	0.064 (6)	−0.012 (5)	0.017 (5)	0.004 (4)

### Geometric parameters (Å, °)

**Table:**

Pb1—Br3	2.8786 (8)	C31—C32	1.365 (14)
Pb1—Br4	2.9927 (7)	N1—H1	0.890
Pb1—Br1	2.9957 (7)	N1—H2	0.890
Pb1—Br6	3.0080 (7)	N1—H3	0.890
Pb1—Br5	3.0095 (7)	N2—H13	0.890
Pb1—Br2	3.1965 (8)	N2—H14	0.890
Pb2—Br8	2.8755 (8)	N2—H15	0.890
Pb2—Br5^i^	2.9935 (6)	N3—H25	0.890
Pb2—Br6	2.9957 (7)	N3—H26	0.890
Pb2—Br1^ii^	3.0082 (7)	N3—H27	0.890
Pb2—Br4^iii^	3.0110 (7)	N4—H37	0.890
Pb2—Br7	3.1982 (8)	N4—H38	0.890
Br1—Pb2^iv^	3.0082 (7)	N4—H39	0.890
Br4—Pb2^iii^	3.0110 (7)	C1—H4	0.970
Br5—Pb2^i^	2.9935 (6)	C1—H5	0.970
N1—C1	1.507 (9)	C2—H6	0.970
N2—C9	1.505 (9)	C2—H7	0.970
N3—C17	1.491 (8)	C4—H8	0.930
N4—C25	1.505 (8)	C5—H9	0.930
C1—C2	1.491 (11)	C6—H10	0.930
C2—C3	1.506 (11)	C7—H11	0.930
C3—C4	1.377 (12)	C8—H12	0.930
C3—C8	1.383 (10)	C9—H16	0.970
C4—C5	1.364 (13)	C9—H17	0.970
C5—C6	1.342 (13)	C10—H18	0.970
C6—C7	1.395 (15)	C10—H19	0.970
C7—C8	1.382 (14)	C12—H20	0.930
C9—C10	1.502 (11)	C13—H21	0.930
C10—C11	1.509 (11)	C14—H22	0.930
C11—C12	1.377 (11)	C15—H23	0.930
C11—C16	1.381 (12)	C16—H24	0.930
C12—C13	1.374 (14)	C17—H28	0.970
C13—C14	1.393 (16)	C17—H29	0.970
C14—C15	1.364 (14)	C18—H30	0.970
C15—C16	1.361 (13)	C18—H31	0.970
C17—C18	1.516 (11)	C20—H32	0.930
C18—C19	1.506 (11)	C21—H33	0.930
C19—C20	1.380 (11)	C22—H34	0.930
C19—C24	1.385 (12)	C23—H35	0.930
C20—C21	1.379 (13)	C24—H36	0.930
C21—C22	1.368 (15)	C25—H40	0.970
C22—C23	1.378 (13)	C25—H41	0.970
C23—C24	1.369 (12)	C26—H42	0.970
C25—C26	1.483 (11)	C26—H43	0.970
C26—C27	1.507 (12)	C28—H44	0.930
C27—C28	1.366 (12)	C29—H45	0.930
C27—C32	1.365 (10)	C30—H46	0.930
C28—C29	1.382 (13)	C31—H47	0.930
C29—C30	1.350 (13)	C32—H48	0.930
C30—C31	1.403 (15)		
			
Br1···N1	3.507 (5)	H10···H44^ix^	3.328
Br1···N1^v^	3.412 (5)	H11···C21^vii^	3.569
Br1···N4	3.564 (5)	H11···C22^vii^	3.047
Br2···N1	3.411 (5)	H11···C23^vii^	3.135
Br2···N3	3.486 (6)	H11···C26^ix^	3.454
Br2···N4	3.462 (5)	H11···C27^ix^	3.563
Br3···N2^iii^	3.393 (5)	H11···C28^ix^	3.551
Br4···N4	3.466 (7)	H11···H23^xii^	3.005
Br4···N4^vi^	3.546 (5)	H11···H34^vii^	3.161
Br5···N3	3.566 (5)	H11···H35^vii^	3.301
Br5···N3^i^	3.473 (6)	H11···H43^ix^	2.654
Br6···N2	3.510 (5)	H11···H44^ix^	3.366
Br6···N2^iii^	3.402 (6)	H12···Br3^v^	3.408
Br6···N3	3.577 (6)	H12···H22^xii^	3.448
Br7···N2	3.416 (5)	H12···H23^xii^	3.596
Br7···N3	3.448 (5)	H12···H35^iv^	3.295
Br7···N4^ii^	3.483 (6)	H12···H36^iv^	2.893
Br8···N1^i^	3.395 (5)	H12···H43^ix^	3.057
N1···Br1	3.507 (5)	H13···Br3^iii^	3.459
N1···Br1^v^	3.412 (5)	H13···Br4^iii^	3.284
N1···Br2	3.411 (5)	H13···Br6	3.171
N1···Br8^i^	3.395 (5)	H13···Br6^iii^	2.604
N2···Br3^iii^	3.393 (5)	H14···Br4^iii^	3.522
N2···Br6	3.510 (5)	H14···Br6	3.209
N2···Br6^iii^	3.402 (6)	H14···Br7	2.544
N2···Br7	3.416 (5)	H15···Br3^iii^	2.596
N3···Br2	3.486 (6)	H15···H42^ii^	3.459
N3···Br5	3.566 (5)	H16···Br6^iii^	3.540
N3···Br5^i^	3.473 (6)	H16···Br8^iii^	2.968
N3···Br6	3.577 (6)	H17···Br2	3.203
N3···Br7	3.448 (5)	H17···Br6	3.166
N4···Br1	3.564 (5)	H18···Br7	3.412
N4···Br2	3.462 (5)	H18···C20	3.150
N4···Br4	3.466 (7)	H18···C21	3.031
N4···Br4^vi^	3.546 (5)	H18···H26	3.259
N4···Br7^iv^	3.483 (6)	H18···H32	3.090
Br1···H1	3.181	H18···H33	2.914
Br1···H1^v^	2.608	H19···Br2	3.547
Br1···H3	3.189	H19···C28	3.128
Br1···H4	3.177	H19···C29	2.968
Br1···H5^v^	3.550	H19···H32	3.371
Br1···H37	3.073	H19···H33	3.240
Br1···H38	3.459	H19···H44	2.833
Br2···H3	2.544	H19···H45	2.545
Br2···H7	3.426	H20···Br8^iii^	3.392
Br2···H8	3.462	H20···H10^x^	3.403
Br2···H17	3.203	H20···H31^x^	3.066
Br2···H19	3.547	H20···H44	2.889
Br2···H27	2.623	H20···H45	3.321
Br2···H30	3.547	H21···C5^viii^	3.116
Br2···H32	3.368	H21···C6^viii^	3.096
Br2···H37	3.426	H21···C18^x^	3.445
Br2···H39	2.726	H21···C19^x^	3.553
Br3···H5^v^	2.971	H21···C24^x^	3.564
Br3···H12^v^	3.408	H21···H9^x^	3.028
Br3···H13^iii^	3.459	H21···H9^viii^	3.243
Br3···H15^iii^	2.596	H21···H10^x^	3.564
Br3···H28^i^	2.961	H21···H10^viii^	3.244
Br3···H41^vi^	3.296	H21···H31^x^	2.649
Br3···H42^vi^	3.486	H21···H36^x^	3.389
Br3···H43^vi^	3.521	H22···C8^viii^	3.521
Br4···H13^iii^	3.284	H22···C23^xi^	3.562
Br4···H14^iii^	3.522	H22···H12^xiii^	3.448
Br4···H37	2.679	H22···H35^xi^	2.747
Br4···H37^vi^	3.273	H22···H36^x^	3.236
Br4···H38^vi^	3.171	H22···H47^viii^	3.200
Br4···H40	3.403	H23···C22^xi^	3.283
Br4···H41^vi^	3.218	H23···C23^xi^	3.442
Br5···H1	3.283	H23···C26^ii^	3.360
Br5···H3	3.524	H23···H11^xiii^	3.005
Br5···H25	3.281	H23···H12^xiii^	3.596
Br5···H25^i^	2.693	H23···H34^xi^	2.678
Br5···H27	3.221	H23···H35^xi^	2.998
Br5···H28^i^	3.380	H23···H42^ii^	2.924
Br5···H29	3.166	H23···H43^ii^	3.107
Br6···H13	3.171	H23···H47^viii^	3.269
Br6···H13^iii^	2.604	H23···H48^ii^	3.409
Br6···H14	3.209	H24···Br7	3.485
Br6···H16^iii^	3.540	H24···C21	3.486
Br6···H17	3.166	H24···C22	3.490
Br6···H25	3.071	H24···H33	3.342
Br6···H27	3.466	H24···H34	3.326
Br7···H4^ii^	3.216	H24···H42^ii^	2.751
Br7···H6^ii^	3.529	H24···H48^ii^	2.887
Br7···H14	2.544	H25···Br5	3.281
Br7···H18	3.412	H25···Br5^i^	2.693
Br7···H24	3.485	H25···Br6	3.071
Br7···H25	3.416	H25···Br7	3.416
Br7···H26	2.706	H26···Br7	2.706
Br7···H38^ii^	2.632	H26···H18	3.259
Br7···H42^ii^	3.545	H27···Br2	2.623
Br7···H48^ii^	3.382	H27···Br5	3.221
Br8···H1^i^	3.444	H27···Br6	3.466
Br8···H2^i^	2.606	H28···Br3^i^	2.961
Br8···H16^iii^	2.968	H28···Br5^i^	3.380
Br8···H20^iii^	3.392	H29···Br5	3.166
Br8···H29^i^	3.284	H29···Br8^i^	3.284
Br8···H30^i^	3.506	H30···Br2	3.547
Br8···H31^i^	3.521	H30···Br8^i^	3.506
Br8···H40^iii^	2.965	H30···C4	3.185
C2···H35^iv^	3.354	H30···C5	3.291
C2···H47	3.372	H30···H2	3.444
C3···H35^iv^	3.594	H30···H8	2.732
C3···H47	3.502	H30···H9	2.951
C4···H30	3.185	H31···Br8^i^	3.521
C4···H47	3.545	H31···C12^ix^	3.178
C5···H9^vii^	3.442	H31···C13^ix^	2.928
C5···H10^vii^	3.513	H31···H9	3.184
C5···H21^viii^	3.116	H31···H20^ix^	3.066
C5···H30	3.291	H31···H21^ix^	2.649
C6···H9^vii^	3.290	H32···Br2	3.368
C6···H21^viii^	3.096	H32···H8	2.925
C6···H44^ix^	3.550	H32···H9	3.415
C7···H43^ix^	2.957	H32···H18	3.090
C7···H44^ix^	3.554	H32···H19	3.371
C8···H22^viii^	3.521	H32···H45	3.580
C8···H35^iv^	3.572	H32···H46	3.429
C8···H43^ix^	3.192	H33···C10	3.380
C10···H33	3.380	H33···C11	3.501
C10···H45	3.380	H33···C16	3.533
C11···H33	3.501	H33···C29^viii^	3.039
C12···H31^x^	3.178	H33···C30^viii^	2.982
C12···H46^viii^	3.473	H33···C31^viii^	3.481
C13···H31^x^	2.928	H33···H18	2.914
C13···H36^x^	3.533	H33···H19	3.240
C13···H46^viii^	3.588	H33···H24	3.342
C13···H47^viii^	3.553	H33···H45	3.082
C14···H35^xi^	3.325	H33···H45^viii^	3.208
C14···H36^x^	3.463	H33···H46^viii^	3.154
C14···H47^viii^	3.037	H34···C15^xi^	3.472
C15···H34^xi^	3.472	H34···C31^viii^	3.564
C15···H35^xi^	3.443	H34···C32^viii^	3.457
C15···H42^ii^	3.294	H34···H11^vii^	3.161
C15···H47^viii^	3.072	H34···H23^xi^	2.678
C16···H33	3.533	H34···H24	3.326
C16···H42^ii^	3.195	H34···H48^ii^	3.483
C16···H47^viii^	3.576	H35···C2^ii^	3.354
C18···H9	3.407	H35···C3^ii^	3.594
C18···H21^ix^	3.445	H35···C8^ii^	3.572
C19···H21^ix^	3.553	H35···C14^xi^	3.325
C20···H10^vii^	3.574	H35···C15^xi^	3.443
C20···H18	3.150	H35···H6^ii^	2.529
C21···H11^vii^	3.569	H35···H11^vii^	3.301
C21···H18	3.031	H35···H12^ii^	3.295
C21···H24	3.486	H35···H22^xi^	2.747
C22···H11^vii^	3.047	H35···H23^xi^	2.998
C22···H23^xi^	3.283	H35···H47^ii^	3.033
C22···H24	3.490	H36···C13^ix^	3.533
C23···H6^ii^	2.944	H36···C14^ix^	3.463
C23···H11^vii^	3.135	H36···H6^ii^	2.833
C23···H22^xi^	3.562	H36···H12^ii^	2.893
C23···H23^xi^	3.442	H36···H21^ix^	3.389
C24···H6^ii^	3.106	H36···H22^ix^	3.236
C24···H21^ix^	3.564	H37···Br1	3.073
C26···H11^x^	3.454	H37···Br2	3.426
C26···H23^iv^	3.360	H37···Br4	2.679
C27···H11^x^	3.563	H37···Br4^vi^	3.273
C28···H11^x^	3.551	H38···Br1	3.459
C28···H19	3.128	H38···Br4^vi^	3.171
C29···H19	2.968	H38···Br7^iv^	2.632
C29···H33^viii^	3.039	H39···Br2	2.726
C29···H45^viii^	3.416	H39···H3	3.582
C29···H46^viii^	3.509	H39···H7	3.217
C30···H8	3.442	H40···Br4	3.403
C30···H33^viii^	2.982	H40···Br8^iii^	2.965
C30···H45^viii^	3.277	H41···Br3^vi^	3.296
C31···H7	3.007	H41···Br4^vi^	3.218
C31···H8	3.489	H42···Br3^vi^	3.486
C31···H33^viii^	3.481	H42···Br7^iv^	3.545
C31···H34^viii^	3.564	H42···C15^iv^	3.294
C32···H7	3.142	H42···C16^iv^	3.195
C32···H34^viii^	3.457	H42···H15^iv^	3.459
H1···Br1	3.181	H42···H23^iv^	2.924
H1···Br1^v^	2.608	H42···H24^iv^	2.751
H1···Br5	3.283	H43···Br3^vi^	3.521
H1···Br8^i^	3.444	H43···C7^x^	2.957
H2···Br8^i^	2.606	H43···C8^x^	3.192
H2···H30	3.444	H43···H11^x^	2.654
H3···Br1	3.189	H43···H12^x^	3.057
H3···Br2	2.544	H43···H23^iv^	3.107
H3···Br5	3.524	H44···C6^x^	3.550
H3···H39	3.582	H44···C7^x^	3.554
H4···Br1	3.177	H44···H10^x^	3.328
H4···Br7^iv^	3.216	H44···H11^x^	3.366
H5···Br1^v^	3.550	H44···H19	2.833
H5···Br3^v^	2.971	H44···H20	2.889
H6···Br7^iv^	3.529	H45···C10	3.380
H6···C23^iv^	2.944	H45···C29^viii^	3.416
H6···C24^iv^	3.106	H45···C30^viii^	3.277
H6···H35^iv^	2.529	H45···H19	2.545
H6···H36^iv^	2.833	H45···H20	3.321
H6···H47	3.229	H45···H32	3.580
H6···H48	3.411	H45···H33	3.082
H7···Br2	3.426	H45···H33^viii^	3.208
H7···C31	3.007	H45···H45^viii^	2.949
H7···C32	3.142	H45···H46^viii^	2.686
H7···H39	3.217	H46···C12^viii^	3.473
H7···H47	2.919	H46···C13^viii^	3.588
H7···H48	3.108	H46···C29^viii^	3.509
H8···Br2	3.462	H46···H8	3.246
H8···C30	3.442	H46···H32	3.429
H8···C31	3.489	H46···H33^viii^	3.154
H8···H30	2.732	H46···H45^viii^	2.686
H8···H32	2.925	H47···C2	3.372
H8···H46	3.246	H47···C3	3.502
H8···H47	3.387	H47···C4	3.545
H9···C5^vii^	3.442	H47···C13^viii^	3.553
H9···C6^vii^	3.290	H47···C14^viii^	3.037
H9···C18	3.407	H47···C15^viii^	3.072
H9···H9^vii^	2.979	H47···C16^viii^	3.576
H9···H10^vii^	2.696	H47···H6	3.229
H9···H21^ix^	3.028	H47···H7	2.919
H9···H21^viii^	3.243	H47···H8	3.387
H9···H30	2.951	H47···H22^viii^	3.200
H9···H31	3.184	H47···H23^viii^	3.269
H9···H32	3.415	H47···H35^iv^	3.033
H10···C5^vii^	3.513	H48···Br7^iv^	3.382
H10···C20^vii^	3.574	H48···H6	3.411
H10···H9^vii^	2.696	H48···H7	3.108
H10···H20^ix^	3.403	H48···H23^iv^	3.409
H10···H21^ix^	3.564	H48···H24^iv^	2.887
H10···H21^viii^	3.244	H48···H34^iv^	3.483
			
Br3—Pb1—Br4	90.86 (2)	C17—N3—H27	109.5
Br3—Pb1—Br1	96.54 (2)	H25—N3—H27	109.5
Br4—Pb1—Br1	88.17 (2)	H26—N3—H27	109.5
Br3—Pb1—Br6	91.58 (2)	C25—N4—H37	109.5
Br4—Pb1—Br6	88.03 (2)	C25—N4—H38	109.5
Br1—Pb1—Br6	171.08 (2)	H37—N4—H38	109.5
Br3—Pb1—Br5	96.46 (2)	C25—N4—H39	109.5
Br4—Pb1—Br5	172.68 (2)	H37—N4—H39	109.5
Br1—Pb1—Br5	91.34 (2)	H38—N4—H39	109.5
Br6—Pb1—Br5	91.41 (2)	C2—C1—H4	109.3
Br3—Pb1—Br2	179.289 (18)	N1—C1—H4	109.3
Br4—Pb1—Br2	88.44 (2)	C2—C1—H5	109.3
Br1—Pb1—Br2	83.56 (2)	N1—C1—H5	109.3
Br6—Pb1—Br2	88.28 (2)	H4—C1—H5	108.0
Br5—Pb1—Br2	84.24 (2)	C1—C2—H6	109.0
Br8—Pb2—Br5^i^	91.00 (2)	C3—C2—H6	109.0
Br8—Pb2—Br6	96.67 (2)	C1—C2—H7	109.0
Br5^i^—Pb2—Br6	88.12 (2)	C3—C2—H7	109.0
Br8—Pb2—Br1^ii^	91.54 (2)	H6—C2—H7	107.8
Br5^i^—Pb2—Br1^ii^	87.98 (2)	C5—C4—H8	118.7
Br6—Pb2—Br1^ii^	170.97 (2)	C3—C4—H8	118.7
Br8—Pb2—Br4^iii^	96.35 (2)	C6—C5—H9	119.7
Br5^i^—Pb2—Br4^iii^	172.63 (2)	C4—C5—H9	119.7
Br6—Pb2—Br4^iii^	91.46 (2)	C5—C6—H10	120.1
Br1^ii^—Pb2—Br4^iii^	91.36 (2)	C7—C6—H10	120.1
Br8—Pb2—Br7	179.356 (18)	C8—C7—H11	120.6
Br5^i^—Pb2—Br7	88.37 (2)	C6—C7—H11	120.6
Br6—Pb2—Br7	83.44 (2)	C7—C8—H12	119.0
Br1^ii^—Pb2—Br7	88.31 (2)	C3—C8—H12	119.0
Br4^iii^—Pb2—Br7	84.28 (2)	C10—C9—H16	109.4
Pb1—Br1—Pb2^iv^	150.77 (3)	N2—C9—H16	109.4
Pb1—Br4—Pb2^iii^	152.07 (3)	C10—C9—H17	109.4
Pb2^i^—Br5—Pb1	152.15 (3)	N2—C9—H17	109.4
Pb2—Br6—Pb1	150.86 (3)	H16—C9—H17	108.0
C2—C1—N1	111.6 (7)	C9—C10—H18	108.7
C1—C2—C3	112.9 (7)	C11—C10—H18	108.7
C4—C3—C8	116.4 (8)	C9—C10—H19	108.7
C4—C3—C2	121.9 (7)	C11—C10—H19	108.7
C8—C3—C2	121.7 (8)	H18—C10—H19	107.6
C5—C4—C3	122.5 (9)	C13—C12—H20	119.4
C6—C5—C4	120.6 (11)	C11—C12—H20	119.4
C5—C6—C7	119.8 (10)	C12—C13—H21	120.9
C8—C7—C6	118.7 (9)	C14—C13—H21	120.9
C7—C8—C3	122.0 (10)	C15—C14—H22	119.4
C10—C9—N2	111.0 (7)	C13—C14—H22	119.4
C9—C10—C11	114.4 (6)	C16—C15—H23	120.4
C12—C11—C16	118.4 (9)	C14—C15—H23	120.4
C12—C11—C10	120.5 (9)	C15—C16—H24	119.2
C16—C11—C10	121.1 (8)	C11—C16—H24	119.2
C13—C12—C11	121.3 (10)	N3—C17—H28	109.5
C12—C13—C14	118.3 (10)	C18—C17—H28	109.5
C15—C14—C13	121.2 (10)	N3—C17—H29	109.5
C16—C15—C14	119.1 (11)	C18—C17—H29	109.5
C15—C16—C11	121.6 (9)	H28—C17—H29	108.1
N3—C17—C18	110.7 (6)	C19—C18—H30	108.5
C19—C18—C17	114.9 (6)	C17—C18—H30	108.5
C20—C19—C24	118.1 (8)	C19—C18—H31	108.5
C20—C19—C18	120.7 (8)	C17—C18—H31	108.5
C24—C19—C18	121.1 (8)	H30—C18—H31	107.5
C21—C20—C19	121.1 (10)	C21—C20—H32	119.4
C22—C21—C20	119.4 (10)	C19—C20—H32	119.4
C21—C22—C23	120.6 (10)	C22—C21—H33	120.3
C24—C23—C22	119.4 (10)	C20—C21—H33	120.3
C23—C24—C19	121.3 (9)	C21—C22—H34	119.7
C26—C25—N4	111.4 (6)	C23—C22—H34	119.7
C25—C26—C27	114.2 (7)	C24—C23—H35	120.3
C28—C27—C32	117.6 (9)	C22—C23—H35	120.3
C28—C27—C26	121.0 (7)	C23—C24—H36	119.3
C32—C27—C26	121.5 (8)	C19—C24—H36	119.3
C27—C28—C29	121.6 (9)	C26—C25—H40	109.4
C30—C29—C28	119.4 (11)	N4—C25—H40	109.4
C29—C30—C31	120.6 (10)	C26—C25—H41	109.4
C32—C31—C30	117.5 (10)	N4—C25—H41	109.4
C27—C32—C31	123.1 (10)	H40—C25—H41	108.0
C1—N1—H1	109.5	C25—C26—H42	108.7
C1—N1—H2	109.5	C27—C26—H42	108.7
H1—N1—H2	109.5	C25—C26—H43	108.7
C1—N1—H3	109.5	C27—C26—H43	108.7
H1—N1—H3	109.5	H42—C26—H43	107.6
H2—N1—H3	109.5	C27—C28—H44	119.2
C9—N2—H13	109.5	C29—C28—H44	119.2
C9—N2—H14	109.5	C30—C29—H45	120.3
H13—N2—H14	109.5	C28—C29—H45	120.3
C9—N2—H15	109.5	C29—C30—H46	119.7
H13—N2—H15	109.5	C31—C30—H46	119.7
H14—N2—H15	109.5	C32—C31—H47	121.2
C17—N3—H25	109.5	C30—C31—H47	121.2
C17—N3—H26	109.5	C27—C32—H48	118.4
H25—N3—H26	109.5	C31—C32—H48	118.4
			
Br3—Pb1—Br1—Pb2^iv^	−71.22 (6)	C9—C10—C11—C12	76.7 (9)
Br4—Pb1—Br1—Pb2^iv^	19.43 (6)	C9—C10—C11—C16	−102.4 (9)
Br5—Pb1—Br1—Pb2^iv^	−167.88 (6)	C16—C11—C12—C13	0.5 (13)
Br2—Pb1—Br1—Pb2^iv^	108.07 (6)	C10—C11—C12—C13	−178.7 (8)
Br3—Pb1—Br4—Pb2^iii^	−89.77 (6)	C11—C12—C13—C14	1.7 (14)
Br1—Pb1—Br4—Pb2^iii^	173.72 (6)	C12—C13—C14—C15	−1.5 (16)
Br6—Pb1—Br4—Pb2^iii^	1.78 (6)	C13—C14—C15—C16	−0.8 (15)
Br2—Pb1—Br4—Pb2^iii^	90.11 (6)	C14—C15—C16—C11	3.0 (14)
Br3—Pb1—Br5—Pb2^i^	−92.15 (6)	C12—C11—C16—C15	−2.9 (13)
Br1—Pb1—Br5—Pb2^i^	4.58 (7)	C10—C11—C16—C15	176.3 (7)
Br6—Pb1—Br5—Pb2^i^	176.10 (6)	N3—C17—C18—C19	−58.9 (9)
Br2—Pb1—Br5—Pb2^i^	87.97 (6)	C17—C18—C19—C20	109.3 (9)
Br8—Pb2—Br6—Pb1	71.35 (6)	C17—C18—C19—C24	−69.1 (9)
Br5^i^—Pb2—Br6—Pb1	−19.43 (6)	C24—C19—C20—C21	−0.8 (12)
Br4^iii^—Pb2—Br6—Pb1	167.92 (6)	C18—C19—C20—C21	−179.2 (7)
Br7—Pb2—Br6—Pb1	−108.01 (6)	C19—C20—C21—C22	1.8 (13)
Br3—Pb1—Br6—Pb2	−76.20 (6)	C20—C21—C22—C23	−1.5 (15)
Br4—Pb1—Br6—Pb2	−167.00 (6)	C21—C22—C23—C24	0.2 (15)
Br5—Pb1—Br6—Pb2	20.30 (6)	C22—C23—C24—C19	0.8 (14)
Br2—Pb1—Br6—Pb2	104.50 (6)	C20—C19—C24—C23	−0.5 (12)
N1—C1—C2—C3	−64.3 (9)	C18—C19—C24—C23	177.9 (7)
C1—C2—C3—C4	103.1 (9)	N4—C25—C26—C27	60.4 (10)
C1—C2—C3—C8	−77.0 (10)	C25—C26—C27—C28	68.7 (11)
C8—C3—C4—C5	0.9 (13)	C25—C26—C27—C32	−111.4 (9)
C2—C3—C4—C5	−179.2 (9)	C32—C27—C28—C29	1.7 (14)
C3—C4—C5—C6	0.9 (16)	C26—C27—C28—C29	−178.4 (9)
C4—C5—C6—C7	−2.5 (17)	C27—C28—C29—C30	0.3 (16)
C5—C6—C7—C8	2.2 (17)	C28—C29—C30—C31	−3.0 (17)
C6—C7—C8—C3	−0.4 (16)	C29—C30—C31—C32	3.7 (16)
C4—C3—C8—C7	−1.1 (13)	C28—C27—C32—C31	−0.9 (14)
C2—C3—C8—C7	179.0 (9)	C26—C27—C32—C31	179.2 (9)
N2—C9—C10—C11	64.1 (9)	C30—C31—C32—C27	−1.8 (16)

Symmetry codes: (i) −*x*+1, −*y*, −*z*; (ii) *x*+1, *y*, *z*; (iii) −*x*+1, −*y*+1, −*z*; (iv) *x*−1, *y*, *z*; (v) −*x*, −*y*, −*z*; (vi) −*x*, −*y*+1, −*z*; (vii) −*x*+1, −*y*, −*z*+1; (viii) −*x*+1, −*y*+1, −*z*+1; (ix) *x*, *y*−1, *z*; (x) *x*, *y*+1, *z*; (xi) −*x*+2, −*y*+1, −*z*+1; (xii) *x*−1, *y*−1, *z*; (xiii) *x*+1, *y*+1, *z*.

### Hydrogen-bond geometry (Å, °)

**Table:**

*D*—H···*A*	*D*—H	H···*A*	*D*···*A*	*D*—H···*A*
N1—H1···Br1	0.89	3.18	3.508 (5)	104
N1—H3···Br2	0.89	2.54	3.411 (5)	165
N2—H13···Br6	0.89	3.17	3.509 (5)	105
N2—H14···Br7	0.89	2.54	3.416 (5)	167
N3—H26···Br7	0.89	2.71	3.448 (6)	142
N3—H27···Br2	0.89	2.62	3.486 (6)	164
N4—H37···Br4	0.89	2.68	3.465 (5)	148
N4—H39···Br2	0.89	2.73	3.462 (6)	140
